# Education Research: The Development and Utilization of a Virtual Twitter Onboarding Curriculum for Neurologists, Trainees, and Students: A Prospective Study

**DOI:** 10.1212/NE9.0000000000200082

**Published:** 2023-07-06

**Authors:** Aaron S. Zelikovich, Joseph E. Safdieh, Matthew S. Robbins

**Affiliations:** From the Department of Neurology, Weill Cornell Medical College, New York, NY.

## Abstract

**Background and Objectives:**

Social media has increased in popularity among neurologists in the past few years without a parallel increase in training opportunities to learn how to use social media effectively. This study tests the feasibility of an asynchronous, virtual onboarding curriculum using Twitter as a tool for professional development for neurologists and neurology trainees.

**Methods:**

Neurologists and neurology trainees were recruited virtually through email, Twitter, and a listserv of the American Academy of Neurology (Synapse). Participants were excluded if they had a professional Twitter account or lived outside the United States. Participants performed all study procedures virtually, including a baseline survey followed by three 30-minute modules: introduction to NeuroTwitter, peer learning, and academic scholarship on Twitter. A postmodule survey was completed to provide postprogram curriculum feedback. Newly created Twitter accounts were followed for 3 months to track Twitter engagement.

**Results:**

Sixty-one participants were screened, and 50 were eligible to enroll. Forty-five (90%) participants completed a consent form and baseline survey. Twenty-seven participants completed all 3 modules, and 26 (52%) completed the postmodule survey. Participants indicated that there was a role for social media in neurology but had minimal to no training on how to use it effectively. Twitter knowledge postmodule completion increased by a median of 2 of 15 questions, with a range of −1 to +5. There were no technical barriers with a virtual-based curriculum, and participants were able to access the modules and surveys successfully. Ninety-six percent of participants would recommend the modules to colleagues. Thirty new Twitter accounts were created with an average of 33 followers, 59 following, 16 tweets, and 61 likes at 4 months.

**Discussion:**

This study highlights the feasibility of virtual asynchronous content leading to an increase in Twitter knowledge among neurologists who completed our modules, though limited by a high dropout rate. Recruitment for virtual asynchronous modules was an effective approach to deliver informative and interactive content for neurologists. Further studies are needed to determine optimal content and length to promote long-term engagement with Twitter.

Social media use has increased in the medical community over the past decade.^1,2^ Twitter has become a platform distinctly used for medical education with the use of hashtags (#) unifying topics making them easily searchable on the platform. Two common hashtags are #FOAM (Free Open Access Medical education) and #MedTweetorials (a thread of multiple tweets linked together to teach a topic or share a case) which allow for widespread and rapid dissemination of high-yield education content. Twitter allows for curated succinct content that fosters the potential for broad engagement. With social media proliferation, physicians are also worried about potential pitfalls, including the protection of patient information, digital professionalism, reputation, and online privacy.^3^ A significant barrier to the greater adoption of a social media platform such as Twitter into medical education is the lack of training on how to use it appropriately and effectively.^4^ There are currently no standards in neurology on how to use social media scholarship for graduate and continuing medical education as well as to measure the effect of an educator or advocate on a platform such as Twitter.^5,6^ Mayo Clinic in Rochester, MN, was one of the first institutions to include social media as part of promotion criteria demonstrating academic advancement.^7^ A group of emergency medicine doctors created a digital impact factor to measure the effect of blogs and podcasts but has yet to be validated in other specialities.^8^

The field of neurology has generally been slow to adopt social media into medical education; however, pilot initiatives have had success. For example, Twitter has been studied in research articles on both Parkinson disease^9^ and dementia^10^ and found to correlate with increased citations and downloads when promoted on social media. Other social media platforms such as Facebook and Instagram also play a role in advertising educational opportunities such as Alzheimer's University^11^ and networking opportunities such as Women Neurologists Group,^4^ but Twitter may have a wider reach for academic scholarship today. Neurology conferences have also engaged participants using Twitter with limited success because the majority of tweets at academic conferences may come from a small percentage of active Twitter users.^12^ Most peer-reviewed journals have Twitter accounts, capitalizing on the opportunity to rapidly disseminate research and other articles to larger audiences beyond their subscribers.^13^ Internal medicine educators use Tweetorials, originally developed in the 2010s by Dr. Tony Breu, as highly effective educational tools.^14,15^ A neurology-specific Tweetorial guide published in 2021 goes through step-by-step on how to create and develop a teaching topic for the beginner in the hopes of encouraging other neurologists to also create their own Tweetorials similar to colleagues in other medical specialties.^16^

Twitter provides a unique opportunity to teach and engage neurologists beyond the traditional mechanisms of textbooks, conferences, and contextual learning in the clinical setting. Social media is not intended to replace but rather supplement and increase the reach of neurology education, networking, and research. Twitter is free, accessible, and available at any time to neurologists and neurology trainees around the world. The asynchronous digital capability allows for educational and research content to transcend a specific location or time as at a conference or grand rounds format, thus increasing the potential reach from hundreds of individuals at an in-person conference or grand rounds to tens of thousands of individuals online. It also supplements in-person advocacy by amplifying neurology initiatives such as American Academy Neurology's Neurology on the Hill (#NOH2023) to show support and increase reach. Neurology residency programs have seen a dramatic increase in the number of residency Twitter accounts since March 2020 to assist in program recruitment for medical students because interviews for neurology residency and fellowship have remained virtual.^17,18^

Despite the many potential benefits in leveraging this digital community, there are no known publicly available resources that provide a curriculum for a neurology-specific audience to learn how to use Twitter effectively. If there were free easily accessible training modules for neurologists to use Twitter, then there would be a lower barrier to greater adoption of Twitter in the neurology community. We created a novel virtual asynchronous module-based curriculum created for neurology attendings, fellows, residents, and neurology-bound medical students to learn the basics of becoming a Twitter user. We hypothesized that participants would gain knowledge and confidence on how to use Twitter and that participation would result in increased Twitter usage and behavior change.

## Methods

### Recruitment

Recruitment occurred between August and December 2021. We recruited participants using institutional review board (IRB)–approved recruitment tweets posted on Twitter from author A.S. Zelikovich's Twitter account (@aszelikovich), posts to neurology communities on the listserv of the American Academy of Neurology (Synapse), and recruitment emails sent to residency program directors. The target recruitment was 50 individuals for feasibility as a pilot study. Eligibility criteria included those who are a United States–based neurologist, neurology trainee, or fourth-year medical student applying to neurology residency and at the time of enrollment lacking a professional Twitter account. Participants were excluded if they had a prior professional Twitter account or lived outside of the United States.

### Modules

Participant screening occurred using a Google form. Eligible participants received a virtual consent form to sign. After consent signature, participants received a deidentified study number and completed a baseline survey that captured participant demographics, a 15-question quiz on Twitter, affiliated neurology department social media presence, and current perceptions (eAppendix 1, links.lww.com/NE9/A36). After the survey, participants were asked to complete 3 modules of 30 minutes in length: introduction to NeuroTwitter, learning from peers on Twitter, and academic scholarship on Twitter. All modules were created by the study team. Modules were prerecorded, on average 30 minutes in length, and hosted on a Google drive with the link sent to participants. Modules could be completed at any time virtually and within 4 weeks of consent completion. After completion of each module, participants completed a 5-question postquiz to assess knowledge acquisition. The quiz questions were identical to those who were asked during the baseline Twitter quiz.

Module 1 discussed the basics of Twitter and the #NeuroTwitter community, provided examples of what a Twitter account looks like, hashtags (#), benefits associated with a Twitter account, and a step-by-step approach to create a new account. Module 2 focused on how to follow other accounts, share content, professionally network, and the “do's and don'ts” of Twitter. Module 3 covered how to promote research, learn about Tweetorials, advocate for patients, and effective highlighting of a training program.

A postmodule survey was sent to individuals who completed all 3 modules to assess the feasibility and effect of the modules (eAppendix 2, links.lww.com/NE9/A37). Participants who created a new professional Twitter account were followed for an additional 3 months to track Twitter activity with the following metrics: number of followers, following, tweets, and likes.

### Twitter Knowledge Quiz

Study authors piloted a 15-question quiz based on their prior experience for topics to cover. Questions included content assessing for hashtags (n = 1 question), website addresses (n = 1 question), Twitter profile elements (n = 1 question), Tweet characters (n = 1 question), interactions (n = 8 questions), Altmetric score (n = 1 question), and Tweetorials (n = 2 questions). The level of complexity of questions was aimed at the novice learner on Twitter. The questions have not been validated, and no quality control was performed.

### Standard Protocol Approvals, Registrations, and Patient Consents

Virtual consent was obtained from all participants through a Google form. IRB approval was obtained through the Weill Cornell IRB under protocol 21-05023611.

### Statistical Analysis

The nature of this study was a pilot study, and therefore, 50 participants were chosen because of feasibility and without powering for statistical significance. Data collected for demographics, baseline survey, postmodule completion, Twitter knowledge, and Twitter engagement at month 4 did not undergo any statistical analysis. Baseline survey questions were collected at a single timepoint. Postmodule completion feedback questions were collected at a single timepoint. Twitter knowledge was tested at baseline, and the same 5 questions were asked after each module for a total of 15 questions. A change in quiz scores was for individual participant scores by taking final total and subtracting the baseline score, with a positive score indicating more questions answered correctly. Pretest and posttest scores for individuals who completed all modules were analyzed with an unpaired *t* test, and *p* values less than 0.05 were considered significant. Twitter engagement data in Figure 5A were reported as absolute number because all participants created a new Twitter account and had zero for all metrics at baseline. A 1-way analysis of variance in GraphPad Prism was performed to compare agree, neutral, and disagree groups in Figure 5B to assess for differences in the number of tweets and likes at 4 months based on answer to question in baseline survey. *p* Values less than 0.05 were reported as statistically significant.

### Data Availability

Deidentified data collected as part of this study in addition to the data published in the manuscript is available by request to any qualified investigator.

## Results

### Participants

**Figure 1:**
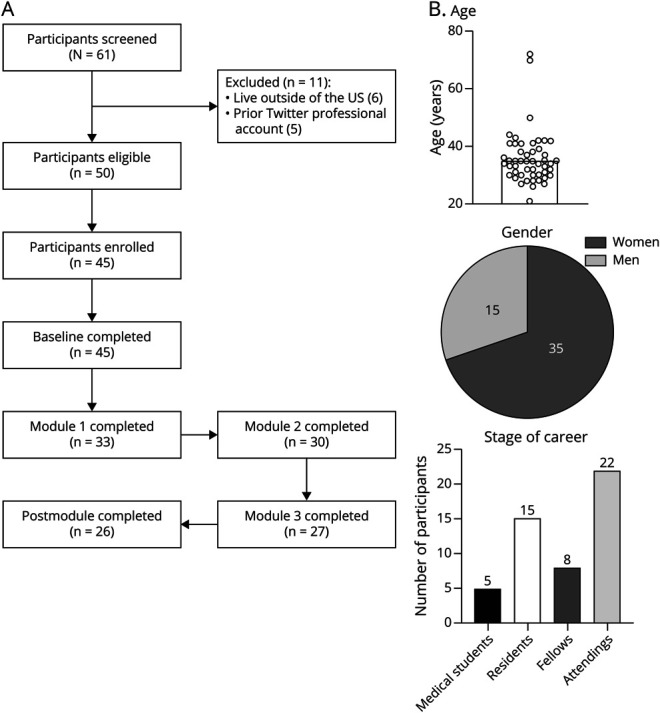
Demographics and Patient Recruitment (A) Sixty-one participants were screened, and 50 were eligible. Forty-five participants signed consent and completed the baseline survey. Thirty-three individuals completed at least 1 module, and 27 completed all 3 modules. (B) The average participant age was 36 years, with a range of 28–72 years. Seventy percent of the participants were women. Attendings made up 44%, residents 30%, fellows 16%, and medical students 10%.

Sixty-one participants completed the screening form. Eleven were ineligible because of living outside the United States (n = 6) or having a prior professional Twitter account (n = 5) (Figure 1A). Of 50 eligible participants, 45 (90%) virtually signed the informed consent form and completed the baseline survey. Twenty-seven (54%) of the eligible participants completed all the modules, and all but 1 completed the postmodule survey. The average participant age was 36 years old, with a range of 21–72 years (Figure 1B). Thirty-five (n = 70%) of the eligible participants were women. Neurology attendings made up the largest proportion of the cohort (44%), and medical students were the fewest (10%). Residents were 30% and fellows 16% of eligible participants.

### Baseline Survey

**Figure 2:**
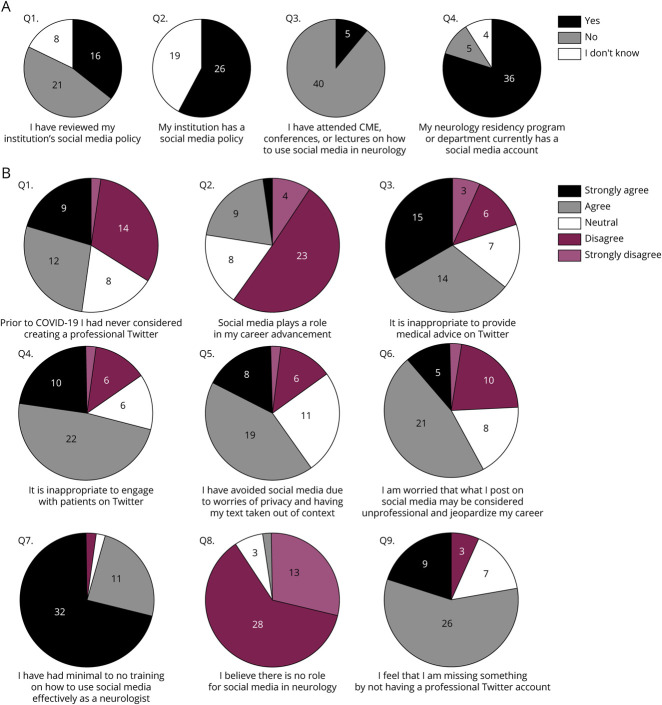
Baseline Survey (A) Forty-five participants completed the baseline survey. Most of the participants had institutional or program social media accounts they were aware of. Very few participants had participated in prior continuing medical education opportunities on using social media in neurology. Approximately one-third of participants have reviewed their institution's social media policy, and no participant stated their institution lacks a social media policy. (B) Ninety-five percent of participants had minimal to no prior training in social media and neurology. Ninety-one percent feel there is some role of social media in neurology. Many participants were concerned that their posts may affect their careers in a negative manner.

Most of the participants (n = 26) reported that they were aware of their institution's social media policy and some have reviewed it (n = 16) (Figure 2A). A small proportion of participants endorsed prior exposure to lectures or conferences pertaining to the use of social media professionally (n = 5). Eighty percent of participants were aware of their institution's social media presence.

Participants overwhelmingly felt that social media has a role in neurology and most had minimal to no training on how to use social media as a neurologist (Figure 2B). Many felt that by not having a professional Twitter account they were missing out on potential benefits. The coronavirus disease 2019 (COVID-19) pandemic encouraged some of the participants to consider joining Twitter professionally. Most of the participants reported that social media is not part of their educator or academic portfolio and career advancement metrics. Participants felt concerned that their tweets or posts may be taken out of context, could be considered unprofessional, and jeopardize their careers.

**Figure 3:**
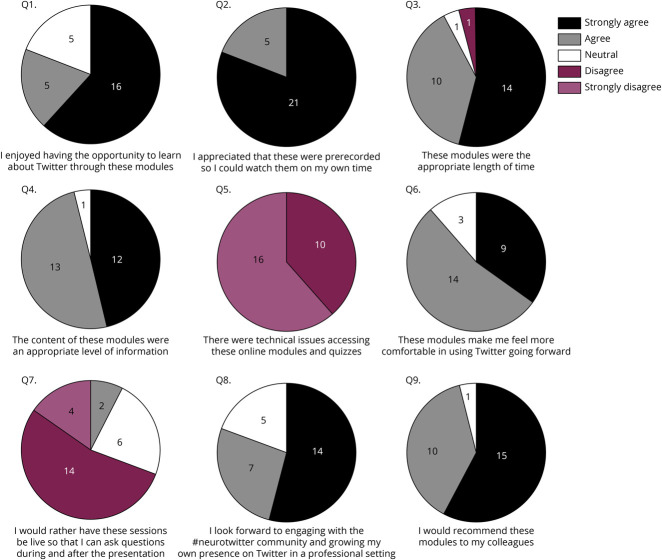
Postmodule Completion Feedback Survey Twenty-six participants completed the postmodule survey. The modules were overall very well received with 80% enjoying learning about Twitter with these modules. The modules were an appropriate length of time, and the content was appropriate. There were minimal technical issues with the preferred prerecorded lectures. All but 1 participant would recommend these modules to their colleagues.

Overall, participants had a good baseline understanding of how Twitter works with the median baseline quiz score of 13 of 15 correct (range 9–15; Figure 3B). The most frequently missed questions related to Tweetorials, Tweet character limits, and Altmetrics (Figure 3A).

### Modules

Thirty-three participants (66%) completed module 1, 30 participants (60%) completed module 2, and 27 (54%) completed module 3 (Figure 1A). Compared with pretest scores (12.8 out of 15), posttest scores (14.4 out of 15) reflected an improvement in most of the participants (81%), with a median score increase of 2, and only 1 participant had a decrease in their scores after completing the modules (Figure 3B).

### Postmodule Survey

**Figure 4:**
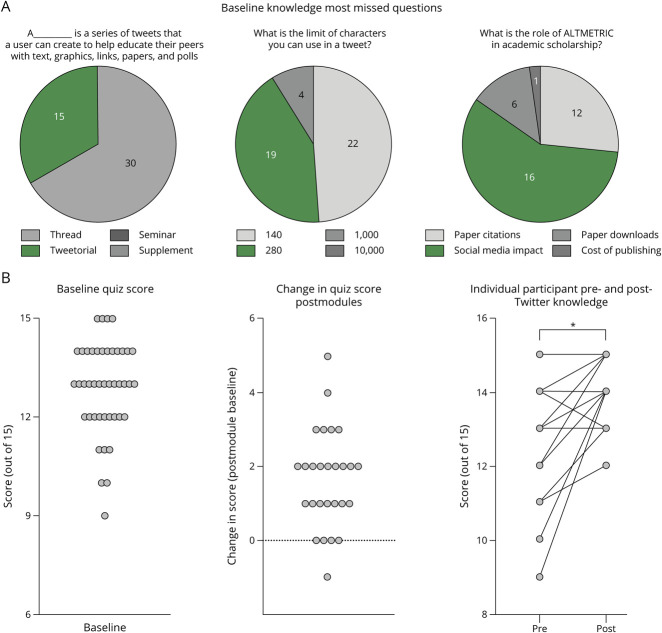
Twitter Knowledge Premodules and Postmodules (A) Forty-five participants completed a baseline survey and pretest quiz. The pretest included 15 questions about Twitter and how to use it. The most frequently missed pretest questions were about tweetorials, the number of characters allowed in a tweet, and the role of altimetric. (B) The 15 baseline questions were repeated after each module (5 after each module). The median premodule score was 13, with a range of 9–15. Twenty-seven participants completed all modules. Twenty-two of 27 participants improved their Twitter knowledge after completing all 3 modules. Posttest scores had a significant improvement from 12.8 to 14.4 (*p* = 0.0001).

Participants overall enjoyed learning about using Twitter professionally and were highly satisfied that they were prerecorded and able to be viewed at any time (Figure 4). Only a few participants (8%) indicated that live sessions would be more beneficial. Participants indicated that the module length of 30 minutes was appropriate and that the content delivered was at a suitable level. There were no technical issues with accessing the videos or postmodule quizzes. Ninety-six percent of participants would recommend these modules to their colleagues, and most of the participants looked forward to engaging on Twitter with the neurology community after completion of the modules.

### Twitter Engagement

Thirty new Twitter accounts were created by those participants who completed module 1. Participants followed accounts that were of interest to them with the median number of Twitter accounts followed as 59 (range of 2–304). There were a few users who were following hundreds of accounts, but the vast majority were below 100. The number of followers was much smaller with a median number of 33 followers (range of 1–195). Only 2 participants had more than 100 followers, and 14 had 10 or fewer followers. Half of the participants never posted a single tweet, and only 7 participants posted more than 10 tweets. Participants had an easier time liking the Tweets of others. Thirteen participants (43%) liked 10 or more tweets, and 5 participants (16%) had more than 100 likes.

**Figure 5:**
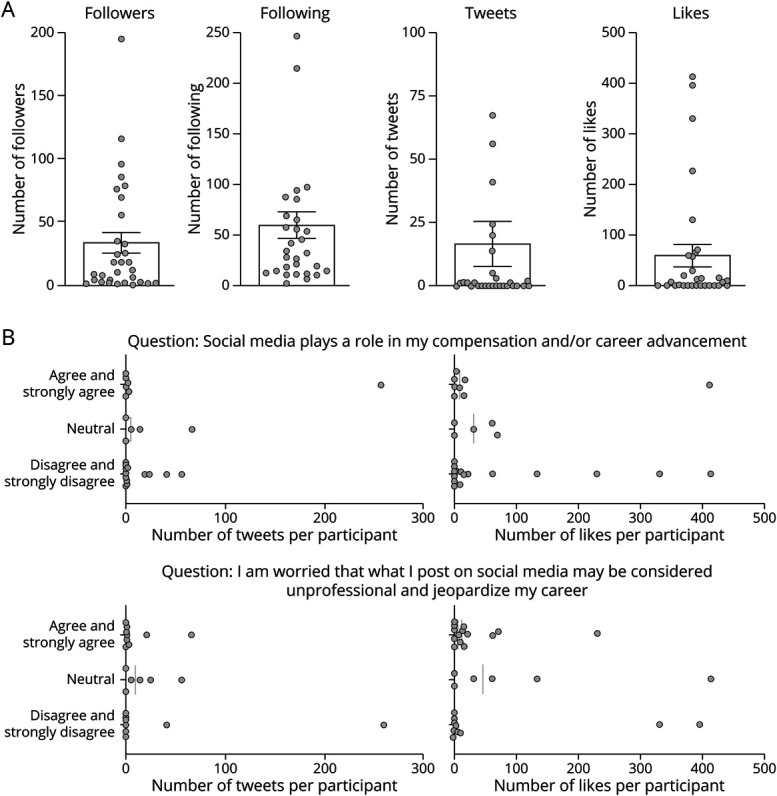
Twitter Engagement at the End of the Study (Month 4) (A) Thirty-three individuals completed module 1, with 2 participants repurposing their personal accounts into professional ones and 1 did not create a new account. Thirty new Twitter accounts were created by study participants. Participants were followed for an additional 3 months after month 1, for a total of 4 months after completion of the baseline survey. The average number of followers was 33, with a range of 1–195. The average amount of following accounts was 59, with a range of 2–304. The average number of tweets was 16, with a range of 0–258. The average number of likes was 61, with a range of 0–412. (B) There was no significant difference in the number of tweets or likes for participants at month 4 based on their response to the baseline survey question for social media playing a role in career advancement or in concern for jeopardizing their career.

In a post hoc analysis to understand long-term engagement, responses to the baseline survey were analyzed for possible factors underlying a low rate of long-term engagement. Two questions were analyzed with long-term Twitter engagement data: (1) “social media plays a role in my compensation and/or career advancement” and (2) “I'm worried that what I post on social media may be considered unprofessional and jeopardize my career.” Participants who agreed that social media plays a role in their compensation had an average of 37 tweets and a median of 0 tweets, whereas participants who disagreed had an average of 8 tweets and a median of 0.5. There was 1 outlier in the agree group with 258 tweets, whereas the rest were between 0 and 3 tweets. The number of likes was similar between both agree and disagree groups with averages of 62 tweets (range of 0–396) for agree and 67 tweets (range of 0–412) for disagree. Participants who worried about tweets jeopardizing their professional career had an average of 7 and a median of 1 tweet(s), whereas those who disagreed had an average of 30 and a median of 0 tweet(s). There was 1 outlier in the disagree group with 258 tweets, the same participant as the outlier in the other question. The number of likes in participants who agreed that posting on Twitter may jeopardize their career was 32 (range of 0–229) likes and those who disagree had 72 (range of 0–396) likes. There was no statistical significance achieved when agree, neutral, and disagree groups were compared for either question (Figure 5B).

## Discussion

Twitter is a unique platform that offers advantages for neurologists to supplement traditional methods of professional development. In 2021, the American Academy of Neurology published a position statement on social media use in health care addressing medical and ethical issues facing neurologists.^19^ This position statement suggested that if used appropriately, social media can enhance the clinical and learning environments of the traditional clinic and hospital settings for research, education, and patient care. Neurologists who engage in clinical or basic science research, medical education, trainee recruitment, and patient advocacy all share in the potential benefit associated with a digital presence to help amplify and disseminate one's work to the greater neurology and public communities. As increasing numbers of neurologists join Twitter, resources need to be created to help inform medical professionals on how to use Twitter safely and effectively. This pilot study using a virtual asynchronous module-based curriculum to onboard neurologists to Twitter was feasible, although there was a high dropout rate.

More specifically, we found many neurologists have no access to educational opportunities to use social media effectively and safely. For some participants, the COVID-19 pandemic was a primary driving force to learn more about Twitter. Most of the participants felt that they were missing out by not having a professional Twitter account but had minimal to no training on how to use it. Asynchronous learning allows a student to learn on their own schedule, thereby increasing flexibility in participating. The benefit of creating virtual asynchronous modules leads to our participants finding them to be easier to complete. The modules provided an appropriate level of content and made those who completed the modules more comfortable in using Twitter professionally. Other online asynchronous studies in neurology clerkship medical student education^20^ and reading electroencephalograms^21^ showed similar feasibility for medical professionals who are time constrained with clinical duties and classes with the benefit of completing the modules on their own time. In our study, 96% of participants felt their colleagues in neurology could benefit from viewing these modules. The average pretest Twitter knowledge score was higher than expected at 13 of 15, with a range of 9–15. It is unclear if this was due to true baseline Twitter knowledge or guessing correctly due to answer choices because each question was required to be answered. Eighty-one percent of participants improved their score suggesting advancement of Twitter knowledge due to completion of the modules. The modules were initially designed for individuals with minimal to no prior Twitter use, but the participants tended to have prior Twitter experience as part of a personal account.

Twitter engagement after 3 months of study module completion failed to show a robust and sustainable behavior change. Half of the participants never posted a single tweet which could be a contributing factor. Module 1 asked participants to create a new Twitter account which 30 of 33 (91%) participants completed. Module 2 asked participants to follow 10 new accounts which lead to a robust response with a median number of 59 Twitter accounts followed, and 28 of 30 accounts (93%) had at least 10 accounts they followed. Module 3 asked participants to post an original tweet, but over half of the participants did not post a single tweet. Unlike modules 1 and 2 which provided a step-by-step guide on how to create a Twitter account and how to like or retweet a tweet, module 3 provided examples of types of tweets but never provided a step-by-step approach to create an original first tweet. The most challenging way to engage on Twitter is to tweet original content, and our modules did not provide enough direction for users to feel comfortable generating an original tweet. Lower levels of engagement with creating a Twitter account and liking other tweets may have been easier for the novice Twitter user, but it is possible that those who felt unprepared to tweet original content felt less engaged on Twitter and thus had a negative effect on long-term behavioral change. In addition, the timeline of 3 months may be too short of a window to observe long-term changes because new Twitter users may need to spend time observing and learning from others before feeling comfortable posting their own original content.

Our pilot study has limitations. First, approximately half of the eligible participants were unable to complete all of the study modules. Multiple factors may have played a role including lack of engagement, high baseline Twitter knowledge, and the voluntary nature of completing the modules. The other difficulty was in recruitment because it may be difficult to reach neurologists, not on Twitter, who may inherently be less likely to engage over email listservs or other forms of communication. We attempted to leverage those neurologists on Twitter already to recruit their colleagues to join. Online forums such as Synapse were used as well as sending emails to neurology residency programs to help recruit trainees, thus limiting the potential pool of neurologists who could participate in this study. The true effect of the learning acquired from the Twitter modules is confounded in the quiz scores for 2 reasons: participants could have randomly guessed correctly on the pretest and inherently individuals will score better on tests taken for the second time. Options for future surveys could include an answer choice of “I don't know” to help decrease guessing bias. Third, this study was a pilot limiting the number of potential participants to 50, and there was no control group for comparison given that participants were new to using Twitter professionally. In Twitter utilization, retweets were not able to be tracked though and could be another marker of engagement in addition to posts and likes.

Future studies are needed to evaluate the long-term efficacy of educational content to engage on social media. Many participants who completed the module portion of the study had minimal to no Twitter engagement after study completion. Certain individuals have greater engagement on Twitter professionally, and it may be useful to incorporate them as case studies into future modules.

## Appendix Authors

**Table:**

Name	Location	Contribution
Aaron S. Zelikovich, MD	Department of Neurology, Weill Cornell Medical College, New York, NY	Drafting/revision of the manuscript for content, including medical writing for content; major role in the acquisition of data; study concept or design; analysis or interpretation of data
Joseph E. Safdieh, MD	Department of Neurology, Weill Cornell Medical College, New York, NY	Drafting/revision of the manuscript for content, including medical writing for content; study concept or design; analysis or interpretation of data
Matthew Stuart Robbins, MD	Department of Neurology, Weill Cornell Medical College, New York, NY	Drafting/revision of the manuscript for content, including medical writing for content; study concept or design; analysis or interpretation of data

## Glossary

COVID-19: coronavirus disease 2019
IRB: institutional review board
